# Outcomes of Mitral Valve Repair for Posterior Leaflet Prolapse, Anterior Leaflet Prolapse, and Bileaflet Prolapse

**DOI:** 10.31083/j.rcm2504146

**Published:** 2024-04-17

**Authors:** Kemin Liu, Qing Ye, Yichen Zhao, Cheng Zhao, Li Song, Yang Liu, Chen Bai, Jie Han, Shengyu Wang, Jiangang Wang

**Affiliations:** ^1^Department of Cardiac Surgery, Beijing Anzhen Hospital, Capital Medical University, 100069 Beijing, China; ^2^Department of Ultrasound, Beijing Anzhen Hospital, Capital Medical University, 100069 Beijing, China; ^3^Department of Pediatric Cardiac Center, Beijing Anzhen Hospital, Capital Medical University, 100069 Beijing, China

**Keywords:** degenerative mitral valve regurgitation, posterior leaflet prolapse, anterior leaflet prolapse

## Abstract

**Background:**

Mitral valve repair (MVr) is an effective treatment for degenerative mitral regurgitation (DMR).And the outcomes and repair rates for posterior leaflet prolapse (PLP), anterior leaflet prolapse (ALP), and bileaflet prolapse (BLP) vary. This study aimed to compare the outcomes of mitral valve repair for patients with PLP, ALP, and BLP.

**Methods:**

From 2010 to 2019, 1192 patients with degenerative mitral valve regurgitation underwent surgery at our hospital. And 1069 patients were identified. The average age of all patients was (54.74 ± 12.17) years old for all patients. 273 patients (25.5%) had ALP, 148 patients (13.8%) had BLP, and 648 patients (60.6%) had PLP. All patients were followed up for an average duration of 5.1 years. We compared the outcomes of patients with ALP, PLP, and BLP.

**Results:**

Patients with ALP were the youngest of the 3 groups and had the highest prevalence of atrial fibrillation. Patients with PLP had the highest prevalence of hypertension, whereas patients with BLP and ALP had larger left ventricular end-diastolic and left ventricular end-systolic diameters. ALP and BLP repairs had a longer cardiopulmonary bypass and aortic cross-clamp time.10 patients dead in-hospital, 5 patients had PLP, 3 had ALP, and 2 had BLP. The 10-year survival cumulative incidences of reoperation among ALP, BLP, and PLP repairs were not significantly different. ALP repair still had higher cumulative incidences of recurrent mitral regurgitation (MR) compared to PLP.

**Conclusions:**

The rates of long-term survival and freedom from reoperation were not significantly different among patients with ALP, BLP, and PLP. ALP repair has higher cumulative incidences of recurrent MR compared to PLP.

## 1. Introduction

Degenerative mitral valve regurgitation is the one of the most common valve diseases in the world [1]. It is generally accepted that mitral valve repair (MVr) is a more preferred choice than mitral valve replacement (MVR) for degenerative mitral regurgitation (DMR) [2, 3], the outcomes and repair rates for posterior leaflet prolapse (PLP), anterior leaflet prolapse (ALP), and bileaflet prolapse (BLP) vary.

Although ALP and BLP repairs are more challenging than PLP repair, studies have demonstrated that PLP repair is more durable than ALP and BLP repairs are [4], prompting some surgeons to favor MVR for ALP and BLP. Compared with developed Western countries, institutions in China adopted MVr considerably later. In addition, comparisons of outcomes between ALP, PLP, and BLP repair are rare. As the second-largest cardiac surgery center in China, we have gained considerable experience regarding degenerative mitral repair. In this study, we retrospectively analyzed patients with degenerative mitral valve regurgitation and compared the outcomes of mitral valve repair for patients with PLP, ALP, and BLP.

## 2. Materials and Methods

### 2.1 Patients

From 2010 to 2019, 1192 patients with degenerative mitral valve regurgitation underwent surgery at our hospital. Patients who were younger than 18 years old, concomitant with congenital heart disease (CHD), and had heart surgery previously were excluded. And 1069 patients were identified. Patients were divided into PLP, ALP, and BLP groups according to their mitral valve pathology.

### 2.2 Surgery

All procedures were performed through a median sternotomy. Aorta and superior and inferior vena cava intubations were used to establish cardiopulmonary bypass. The mitral valve was exposed through a right atrium, atrial septal, or atrial sulcus incision. Before surgery, TEE was performed conventionally to further explore the structure of MV, help us better establish the surgical plans.

PLP was corrected through quadrangular or triangular resection and involved the use of the sliding technique if further resection was needed. Due to the importance of the recent “respect rather than resect” principle, the leaflet folding and polytetrafluoroethylene chordae were also adapted, especially for patients with isolated P2 prolapse. For commissural prolapse, the commissural closure or folding was used.

ALP repair is considered more challenging than PLP repair. Therefore, for patients with ALP, polytetrafluoroethylene chordae, commissural closure and triangular resection were adopted. Additionally, the size of annuloplasty ring or band was according to the surface area of the anterior leaflet.

Transoesophageal echocardiography was routinely performed to evaluate the quality of the repair. If there were residual moderate MR, it would be re-repaired.

Othe procefures were also performed as required.

### 2.3 Follow-Up

The patients were followed up at 3, 6, and 12 months, and then annually. The outcomes include the long term survival, recurrent mitral valve regurgitation and mitral valve reoperation. The degree of regurgitation was classified as mild (effective regurgitate orifice area (EROA) <0.2 ${\mathrm{cm}}^{2}$), moderate (0.2–0.39 ${\mathrm{cm}}^{2}$), or severe (≥0.4 ${\mathrm{cm}}^{2}$) [5]. Patients with more than moderate mitral valve regurgitation were considered to have recurrent mitral valve regurgitation. The pulmonary hypertension was diagnosed by the pulmonary artery systolic pressure (PASP) measured by the echocardiography using the modified Bernoulli equation on the transtricuspid continuous-wave Doppler signal while adding right atrium (RA) pressure. And patients were divided into 3 groups: normal pulmonary hypertension (PH), mild PH (35 to 44 mmHg), moderate PH (45 to 59 mmHg) and severe PH (≥60 mmHg) [6].

### 2.4 Statistical Analyses

Data are presented as mean ± SD for continuous variables while the differences among groups were expressed using the the analysis of variance (ANOVA). For categorical variables, data are presented as frequencies and percentages, and the differences among groups were tested using the chi-square analysis (Pearson).

Cox’s proportional hazard regression model was used to evaluate the effects of multiple potential factors on long-term survival. Kaplan–Meier analysis was used to assess the differences in survival among the groups. The log-rank test determined statistical significance among the risk categories for all Kaplan–Meier analyses. Data analysis was performed using SPSS version 22 (SPSS, Inc. Chicago, IL, USA) and R 3.6.1 using the cmprsk package (R Foundation for Statistical Computing, 169 Vienna, Austria).

## 3. Results

### 3.1 Perioperative Data

In total, 1069 patients were identified in this study, among whom 273 (25.5%) had ALP, 148 (13.8%) had BLP, and 648 (60.6%) had PLP. A total of 90 patients underwent MVR, among whom 27 had ALP, 30 had PLP, and 33 had BLP. The total repair rate was nearly 92%, with 91% for ALP, 95% for PLP, and 82% for BLP. Patients with ALP or BLP had a higher probability of repair failure than patients with PLP.

60 patients were considered to have Barlow’s disease. Most patients with ALP (65.7%) were female, while males constituted the majority of patients with ALP (35.5%) and BLP (35.1%). Patients with PLP were the oldest (56.30 ± 10.96 years old), followed by patients with BLP (50.46 ± 14.09 years old) and ALP (53.33 ± 13.08 years old). Patients with PLP and ALP had the highest prevalence of hypertension and atrial fibrillation (AF), respectively. Patients with PLP had the smallest left atrial diameter and left ventricular end-diastolic and end-systolic diameters (Table 1).

**Table 1.** **Baseline characteristics of patients**.

Variables		PLP (n = 648)	ALP (n = 273)	BLP (n = 148)	*p*-value
Female gender		426 (65.7%)	97 (35.5%)	52 (35.1%)	0.927
Age		56.30 ± 10.96	53.33 ± 13.08	50.46 ± 14.09	<0.001
BMI		24.97 ± 3.29	24.23 ± 3.49	24.22 ± 3.63	0.002
NYHA class					0.162
	I	12 (2%)	9 (3.3%)	8 (5.4%)	
	II	434 (67%)	175 (64.1%)	103 (69.6%)	
	III	189 (29%)	83 (30.4%)	33 (22.3%)	
	VI	13 (2%)	6 (2%)	4 (3%)	
Hypertension		282 (44%)	90 (33%)	46 (31%)	0.001
AF		192 (30%)	108 (40%)	44 (30%)	0.010
CAD		67 (10%)	23 (8%)	12 (8.1)	0.542
DM		54 (8%)	12 (4%)	9 (6%)	0.091
CVD		26 (4%)	12 (4%)	4 (23)	0.684
Echocardiographic data					
	LAD (mm)	46.50 ± 8.95	48.34 ± 9.73	46.80 (10.71)	0.026
	LVEDD (mm)	56.31 ± 6.70	57.56 ± 7.51	57.57 ± 7.55	0.018
	LVESD (mm)	36.38 ± 5.70	37.83 ± 6.83	37.18 ± 6.21	0.003
EF (%)		63.14 ± 6.53	62.21 ± 7.08	63.17 ± 7.00	0.141
Peak E wave velocity		124.85 (35.65)	120.58 (37.37)	124.66 (36.85)	0.443
More than moderate TR		164 (25%)	86 (32%)	27 (18%)	0.011
PHT		308 (47%)	145 (53%)	89 (60%)	0.130
	Mild	213 (33%)	91 (33%)	50 (34%)	
	Moderate	68 (10%)	27 (10%)	9 (6%)	
	Severe	27 (4%)	10 (4%)	0 (0%)	

PLP, posterior leaflet prolapse; ALP, anterior leaflet prolapse; BLP, bileaflet prolapse; BMI, body mass index; NYHA, New York Heart Association; AF, atrial fibrillation; CAD, coronary heart disease; DM, diabetes mellitus; CVD, cerebral vascular disease; LAD, left atrial diameter; LVEDD, left ventricular end-diastolic diameter; LVESD, left ventricular end-systolic diameter; EF, ejection fraction; TR, tricuspid regurgitation; PHT, pulmonary hypertension.

### 3.2 Surgery Data

The Maze procedure was more common for ALP repair. BLP repair had longer cardiopulmonary bypass (CPB) and aortic cross-clamp durations than PLP and ALP repairs (*p* < 0.05). 10 patients died in-hospital, 5 patients had PLP, 3 had ALP, and 2 had BLP (Table 2).

**Table 2.** **Procedures data**.

Variables	PLP (n = 648)	ALP (n = 273)	BLP (n = 148)	*p*-value
Other procedures				
TVP	424 (65%)	188 (69%)	98 (66%)	0.601
Maze procedure	181 (28%)	101 (37%)	40 (27%)	0.016
AVR	37 (6%)	19 (7%)	8 (5%)	0.728
CABG	46 (7%)	17 (6%)	8 (5%)	0.719
CPB time	104.90 ± 41.63	112.37 ± 38.68	124.99 ± 46.82	<0.001
Cross-clamp time	72.96 ± 29.32	81.67 ± 31.57	92.34 ± 35.86	<0.001

PLP, posterior leaflet prolapse; ALP, anterior leaflet prolapse; BLP, bileaflet prolapse; TVP, tricuspid valvuloplasty; AVR, aortic valve replacement; CABG, coronary artery bypass grafting; CPB, cardiopulmonary bypass.

The surgical techniques were shown in Table 3 detail. The average size of the annuloplasty ring was 32 mm. The Carpentier-Edwards Physio I/II Semirigid Annuloplasty Rings were most frequently used (n = 874). The Sorin Memo 3D Semirigid Annuloplasty Rings (n = 169) and Medtronic CG FUTURE annuloplasty rings (n = 26) were also used [7].

**Table 3.** **Surgical techniques**.

Surgical techniques	PLP (n = 648)	ALP (n = 273)	BLP (n = 148)	*p*-value
Leaflet resection	354 (55%)	2 (0.7%)	40 (3%)	<0.001
Chordal replacement	75 (12%)	185 (68%)	83 (56%)	<0.001
Leaflet folding	250 (39%)	47 (17%)	50 (34%)	<0.001
Edge-to-edge repair	7 (1%)	46 (17%)	35 (24%)	<0.001
Chordal shortening	21 (3%)		1 (0.7%)	<0.001
Chordal transfer		10 (4%)	2 (1%)	<0.001
Commissural closure	7 (1%)	20 (7%)	18 (12%)	<0.001
Annuloplasty ring size	31.76 ± 1.69	31.95 ± 0.66	32.81 ± 2.50	<0.001
Carpentier-Edwards Physio I/II	571 (88%)	183 (67%)	120 (81%)	<0.001
Sorin Memo 3D	71 (11%)	70 (26%)	28 (19%)	<0.001
Medtronic CG FUTURE	6 (1%)	20 (7%)	0 (0%)	<0.001

PLP, posterior leaflet prolapse; ALP, anterior leaflet prolapse; BLP, bileaflet prolapse.

Table 4 shows the echocardiographic data before patient discharge. The left atrial diameter, left ventricular end-diastolic diameter; left ventricular end-systolic diameter; and ejection fraction was not significantly different between the groups.

**Table 4.** **Echocardiographic data before patient discharge**.

Variables	PLP (n = 648)	ALP (n = 273)	BLP (n = 148)	*p*-value
Echocardiographic data				
LAD (mm)	38.15 ± 7.02	38.00 ± 7.53	37.59 ± 7.51	0.402
LVEDD (mm)	48.62 ± 5.35	49.07 ± 6.13	49.04 ± 6.46	0.473
LVESD (mm)	33.21 ± 5.71	33.81 ± 5.90	33.55 ± 6.51	0.342
EF (%)	58.98 ± 7.64	58.82 ± 7.14	58.78 ± 7.42	0.736
More than mild MR	18	14	8	0.306
PHT	15	18	4	0.228

PLP, posterior leaflet prolapse; ALP, anterior leaflet prolapse; BLP, bileaflet prolapse; LAD, left atrial diameter; LVEDD, left ventricular end-diastolic diameter; LVESD, left ventricular end-systolic diameter; EF, ejection fraction; MR, mitral regurgitation; PHT, pulmonary hypertension.

### 3.3 Survival

During a mean follow-up of 5 years (5–7 years), Nearly 5% (53/1059) of patients were lost during follow-up. And 33 patients died, among whom 23 had PLP, 6 had ALP, and 4 had BLP. Cardiac deaths totaled 14 in the PLP group due to heart failure (n = 9), myocardial infarction (n = 2), sudden unexplained death (n = 2), and infective endocarditis (n = 1); 5 in the ALP group due to heart failure (n = 1), sudden unexplained death (n = 1), and infective endocarditis (n = 1); and 2 in the BLP group due to heart failure (n = 1) and infective endocarditis (n = 1).

**Fig. 1.**
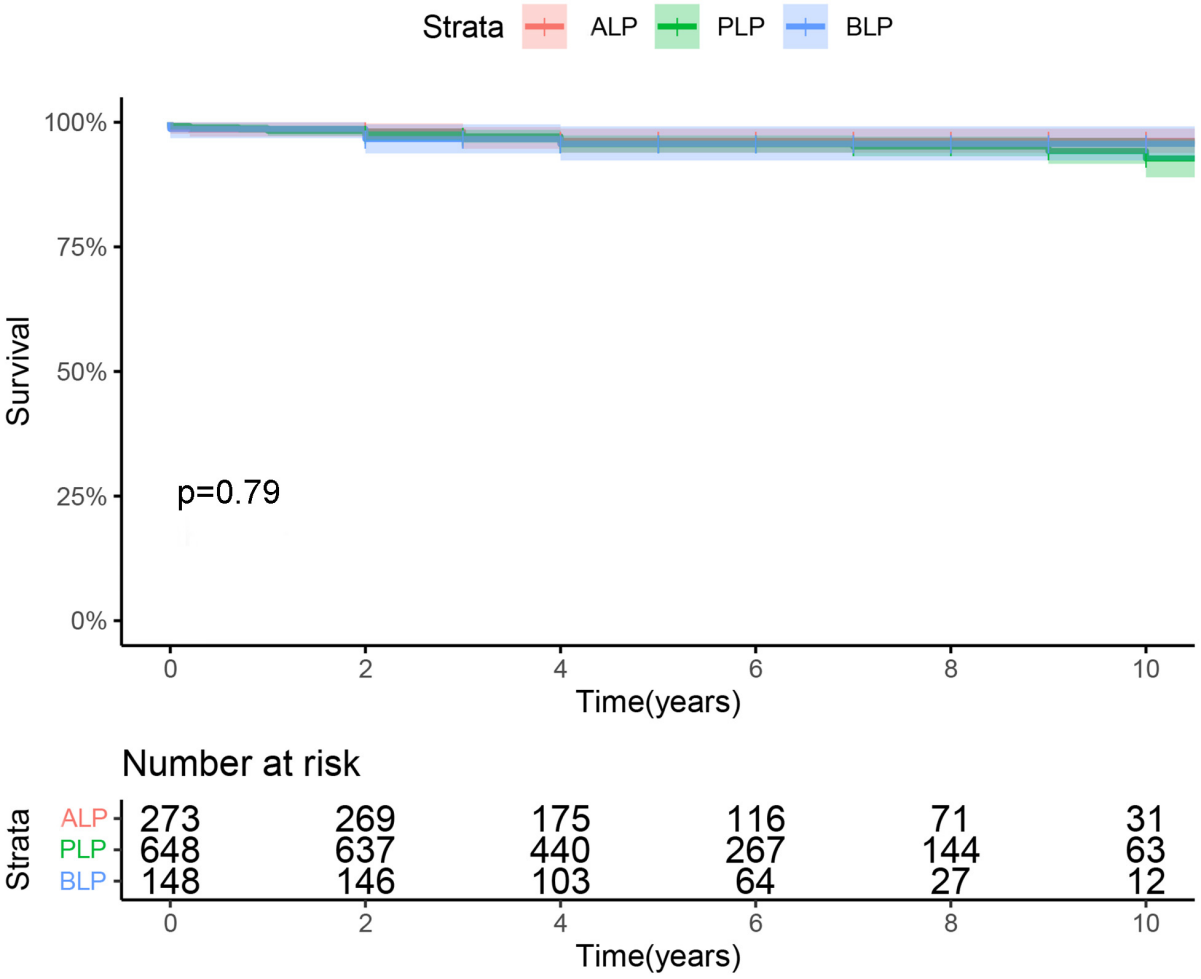
**Overall survival for isolated anterior, bileaflet, and posterior leaflet prolapse repair**. BLP, bileaflet prolapse; ALP, anterior leaflet prolapse; PLP, posterior leaflet prolapse.

Fig. 1 shows the long-term survival of patients with ALP, BLP, and PLP. The 10-year overall survival was 93 ± 3% for patients with PLP and 96 ± 1% for patients with ALP or BLP, which was not significantly different between the groups.

### 3.4 Reoperation and Recurrent MR 

**Fig. 2.**
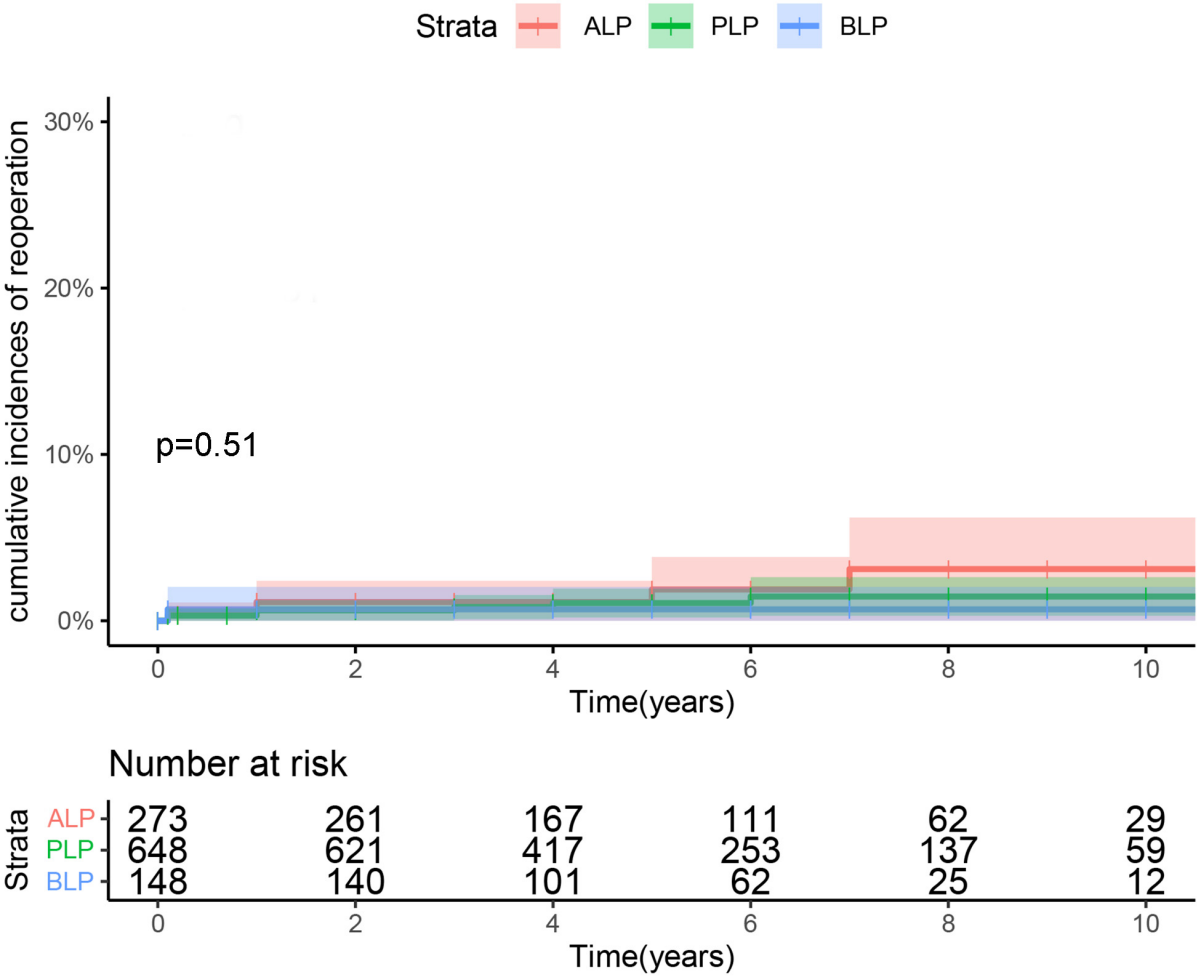
**Cumulative incidence of reoperation for isolated anterior, bileaflet, and posterior leaflet prolapse repair**. BLP, bileaflet prolapse; ALP, anterior leaflet prolapse; PLP, posterior leaflet prolapse.

Overall, 13 patients underwent reoperation, among whom 7 had PLP, 5 had ALP, and 1 had BLP. The long-term freedom from reoperation rates were 96.8 ± 1.6%, 98.5 ± 0.6%, and 99.5 ± 0.5% for PLP, ALP, and BLP, respectively (Fig. 2).

119 patients had mitral valve regurgitation during follow up , including 82 patients with moderate MR and 37 patients with severe MR, among whom 61 had PLP (19 had severe MR), 42 had ALP (11 had severe MR), and 16 had BLP (7 had severe MR). The long-term freedom from recurrent MR rates for PLP, ALP, and BLP was 72.4 ± 4.7%, 85.2 ± 2.4%, and 82.7 ± 3.6%, respectively. After adjusting for confounding variables via the competitive risk model, BLP repair resulted in comparable long-term durability to ALP and PLP repairs. Notably, however, PLP repair had better durability than ALP repair.

**Fig. 3.**
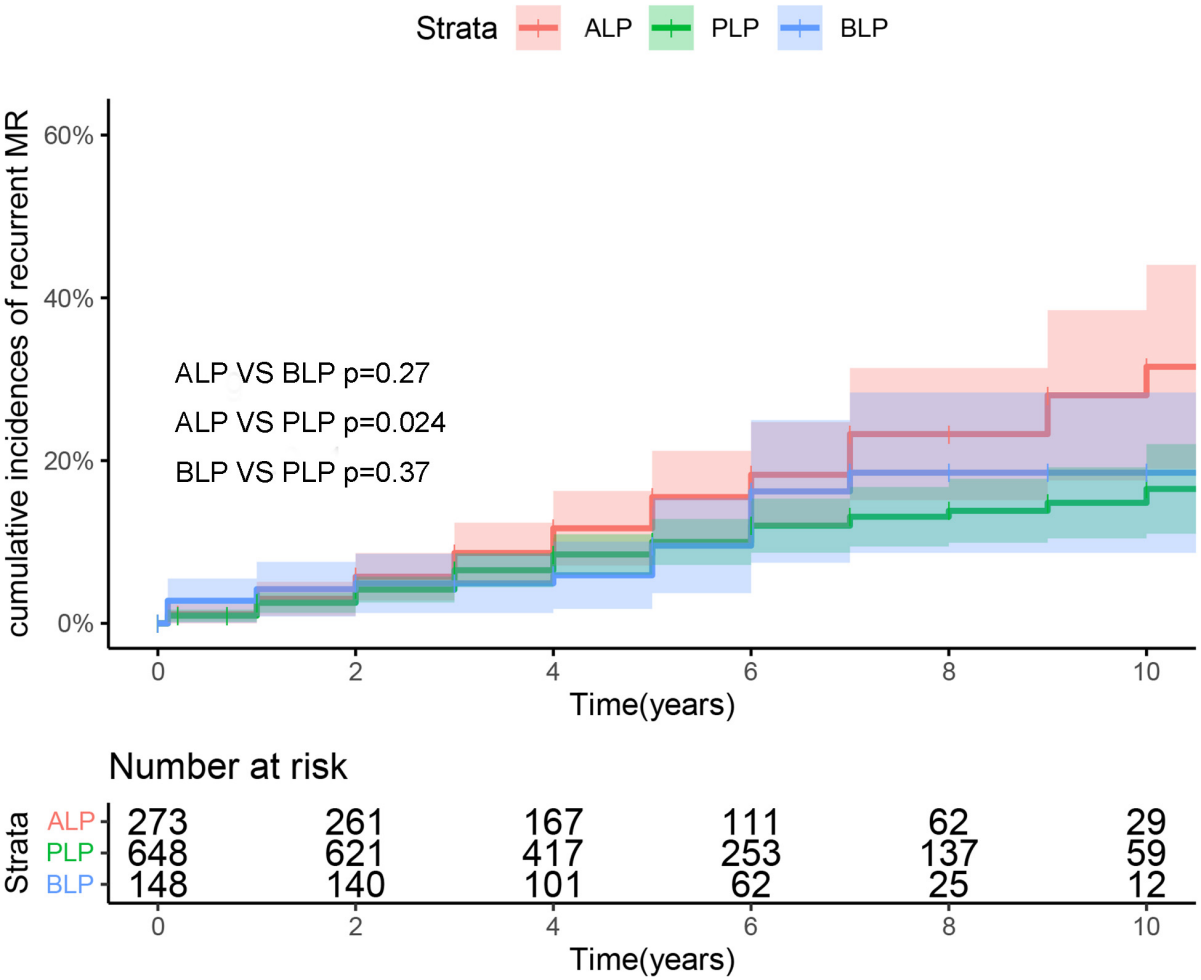
**Cumulative incidence of recurrent mitral regurgitation for isolated anterior, bileaflet, and posterior leaflet prolapse repair**. BLP, bileaflet prolapse; ALP, anterior leaflet prolapse; MR, Mitral regurgitation; PLP, Posterior leaflet prolapse.

Fig. 3 shows the cumulative incidences of recurrent MR between PLP, ALP, and BLP. ALP may be a greater risk factor for long-term recurrent MR compared to PLP.

In our institution, patients were asked to have echocardiography 1 month, 6 months and 1 year after operation at our hospital. If there was nothing wrong, patients could have echocardiography once a year at the local hospitals. And 787 patients had undergone echocardiography, and 467 patients underwent echocardiography in our hospital.

Table 5 shows that the pathology of mitral valve is not a risk factor of long-term survival. Table 6 shows that after adjusting for confounding variables via the competitive risk model, the BLP repair had a comparable long-term durability to ALP and PLP repair. Notably, however, PLP repair had better long-term durability than ALP repair.

**Table 5.** **Cox regression analysis of survival**.

	Univariate analysis		Multivariate analysis	
	HR (95% CI)	*p*-value	HR (95% CI)	*p*-value
Age	1.05 (1.01–1.08)	0.019	1.04 (1.00–1.08)	0.047
Atrial fibrillation	2.30 (1.08–4.91)	0.030		
LVESD >40 mm	2.48 (1.17–5.28)	0.018		
EF <60%	3.46 (1.62–7.39)	0.001	3.09 (1.43–6.65)	0.040
Leaflet prolapse (PLP)				
ALP	1.30 (0.61–2.76)	0.49		
BLP	1.23 (0.44–3.46)	0.69		
Maze surgery	2.60 (1.22–5.53)	0.013		

HR, hazard ratio; CI, confidence interval; EF, ejection fraction; LVESD, left ventricular end-systolic diameter; ALP, anterior leaflet prolapse; BLP, bileaflet prolapse; PLP, posterior leaflet prolapse.

**Table 6.** **Risk factor analysis of recurrent MR using the competitive risk model**.

	Univariate analysis		Multivariate analysis	
	HR (95% CI)	*p*-value	HR (95% CI)	*p*-value
Age	1.02 (1.00–1.03)	0.034	1.02 (1.00–1.03)	0.047
Sex	1.65 (1.15–2.36)	0.006	1.55 (1.08–2.22)	0.017
BMI	0.92 (0.88–0.97)	0.003	0.93 (0.89–0.98)	0.0082
Atrial fibrillation	1.41 (0.98–2.03)	0.063		
E wave velocity	1.01 (1.00–1.01)	0.003	1.01 (1.00–1.01)	0.0046
Leaflet prolapse (PLP)				
ALP	1.75 (1.18–2.60)	0.0057	1.640 (1.07–2.52)	0.024
BLP	1.28 (0.79–2.08)	0.310	1.34 (0.831–2.16)	0.230
Pulmonary hypertension	1.32 (1.11–1.58)	0.002		
Residual MR post-surgery	1.87 (1.21–2.90)	0.005	1.68 (1.08–2.62)	0.020
CPB time	1.00 (1.00–1.01)	0.034		
Aortic clamping time	1.00 (1.00–1.01)	0.031		

HR, hazard ratio; CI, confidence interval; BMI, body mass index; PLP, posterior leaflet prolapse; ALP, anterior leaflet prolapse; BLP, bileaflet prolapse; MR, mitral regurgitation; CPB, cardiopulmonary bypass.

## 4. Discussion

In this retrospective study, we found that the rates of long-term survival and freedom from reoperation did not differ between ALP, BLP, and PLP. Compared to PLP repair, ALP had a higher probability of having recurrent MR (*p* < 0.05). BLP repair had similar durability to ALP and PLP repair.

Degenerative mitral valve disease is one of the most common heart valve diseases in the world. Outcomes among ALP, PLP, and BLP repair have been compared for years. Mohty *et al*. [8] may have been the first to analyze a large series of patients who underwent MVr and report their long-term follow-up results. They found that the reoperation rate was higher for ALP than for PLP. David *et al*. [9] also reported that the results of PLP repair were better than those of both ALP and BLP repairs. However, the outcomes of ALP repair have also improved with the increase in its usage and the development of repair technology. Castillo *et al*. [10] reported that they could achieve a near 100% repair rate, regardless of ALP or BLP, while Bonis *et al*. [11] demonstrated similar long-term results regarding MVr for ALP and PLP. All patients had similar survival and reoperation rates; however, the rate of freedom from recurrent MR was not compared.

The safety and efficacy of MVr for DMR have already been confirmed. However, the durability of ALP, PLP, and BLP repair remains unclear. Brescia *et al*. [12] conducted a retrospective, propensity-matched analysis and found that the long-term survival and reoperation rates, as well as the MR grade, were not significantly different between the two groups. Here, we conducted this retrospective investigation to compare the outcomes of ALP and PLP repair. We found that if we divided the patients into a complex group (ALP and BLP) and a simple group (PLP), after adjusting confounding factors with the competitive risk model, the long-term durability between the two groups was not significantly different (*p* > 0.05; Table 5). Previous studies also found that BLP repair was more durable than ALP repair and that ALP may be an independent risk factor for recurrent MR [9, 13]. Nevertheless, we found that the durability of ALP and BLP repairs was comparable (Fig. 3). Moreover, when comparing ALP, BLP, and PLP repair, we found that ALP repair had a higher probability of having recurrent MR compared to PLP repair and that BLP repair had similar durability compared to ALP and PLP repairs (Table 6). We found a similar durability between BLP and PLP repairs, as reported previously [14, 15]. This might have occurred due to an inadequate number of patients with BLP (n = 148) and BLP having the lowest rate of repair (82%).

PLP repair has been standardized and has demonstrated excellent long-term outcomes [16]. In contrast, ALP and BLP repairs are considered more challenging and diverse. Carpentier’s technique [17], the edge-to-edge technique, and chordal replacement with polytetrafluoroethylene sutures have all been used for the repair of ALP and BLP. As the second largest cardiac surgery center in China, we have gained considerable experience in ALP repair. In our experience, Carpentier’s technique combined with chordal replacement is an effective method of ALP repair, and the edge-to-edge technique may be a useful rescue technique for failed repair [18]. Earlier, we used to perform some edge-to-edge techniques for ALP repair, and most patients had recurrent MR during follow-up. Nowadays, artificial chordal implantation is the basic measure of ALP repair. Finding the suitable length and number of neochordaes is the key point in performing this procedure. Briefly, the TEE during operation measures the distance between the papillary muscle and the coaptation with the normal leaflet, which guides us to estimate the length of the neochordae. Consequently, the neochordae are placed between the papillary muscle and the free margin of the leaflet. The length can then be adjusted using a forceful saline injection into the left ventricle. Valve competence is evaluated at the same time. As such, 2–3 neochordae may be suitable for ALP repair, but BLP repair may need more. Based on this procedure, we suspect that the dimension of the left ventricle might have decreased postoperatively, leading to an unsuitable length of the neochordae. Eventually, this process is likely to cause recurrent MR and may explain why ALP had low durability.

It is generally accepted that MVr is superior to MVR for degenerative MR [19], and guidelines recommend early surgery for patients with preserved heart function [20]. However, early surgery for patients with ALP and BLP is challenging [21], and outcomes vary among different hospitals [22, 23]. Our results revealed that ALP and BLP repairs can yield excellent results. Some patients may have an imperfect repair with more than mild MR after the operation, and some may develop moderate and severe MR years later. Therefore, surgeons need to be more careful when performing MVr and pay more attention to the TEE results. Early surgery should be recommended only with durable repairs.

### Limitations

This is a retrospective observational study performed at a single center. The sample size is not large enough, especially for the patients with BLP, which may cause bias. The longest period of follow-up is 10 years, we are unabled to obtain very long-term outcomes.We also can not obtain all patients’ echocardiograms during follow-up, which may impact assessment of the duribiity of mitral valve repair. This retrospective observational study is the summary of our previous jobs. And prospective research about DMR is also underway.

## 5. Conclusions

Degenerative MR repair can achieve excellent results. The rates of long-term survival and freedom from reoperation were not significantly different among ALP, BLP, and PLP patients. ALP repair still has higher cumulative incidences of recurrent MR compared to PLP. For these patients, surgeons should be more careful.

## Data Availability

All relevant data are provided in the manuscript.
